# One on Dr. Daniel

**Published:** 1896-02

**Authors:** 


ONE ON DR. DANIEL.—Dr. Merriman,—the JOURNAL'S genial philosopher, who occasionally brings sunshine into the dreary sanctum of ye hard-worked editor,—dropped in one day last week—as fat and as jolly as ever. (He is kind enough to say he *has* to come in once a month to "load up,"—on what, he says not;—like the cars that carry the storage batteries—have to go to the dynamo for their supply of lightning, we suppose. We feel that the compliment is on the other side.) However, be that as it may—a kind of mutual admiration exists between the office and the philosopher.

Without any ceremony, the doctor sat down, and began, in *medias res*, "Hudson," he said—(Hudson was closely engaged in footing up expense account, to see if he could make it come inside of receipts,—I was laboring on a manuscript that would have discounted Horace Greeley's worst specimen,—Bennett was writing a love letter,—while the office boy was whistling, "Henrietta—have you met her," keeping time by a tattoo with both hands and both feet)—"Hudson," said the doctor, "I've got a good one on Dan'els";—and here he chuckled till the shovel and tongs and the other costly office furniture rattled. "You know Dan'els is a great dermatologist (I don't think),—got a big reputation for skin diseases—down at the Wallow, any way. I've got a case of skin trouble down there that's pestering me, and after I had done for him everything *I* knew, I brought him up here to consult Dan'els.—I thought it was eczema, and treated it as such; told Dan'els I thought so. Well, the patient,—his name is Skaggs,—he is a sorry lookin' cuss, —said he had scratched till he was paralyzed in both arms. He rolled up his sleeves and his britches legs, and Dan'els put on his specs and examined it carefully,—asking him some questions. Then he raised up, and removing his eye glasses,—said, impressively, and in that grand oracular manner he has,—emphasizing with his fore-finger—'It's *psoriasis*, doctor; *psoriasis gyrata;*—a well marked case—a beautiful case! You see, doctor, the distinguishing features are—the uniform elevated areas of infiltrated tissue,—and the enclosed areas of sound skin—and the uniform redness—and the persistent dryness; but more than *all*—its occurrence ONLY on the extensor surfaces. Now, you see, doctor, this man has it on the extensors of arms and legs, and on his back;—the absence of it on the breast and abdomen'—here, turning to Skaggs, he said: 'Never had it on your belly, did you, Skaggs?' 'Belly, nothing,' said that individual. 'Why, doc., hits *all over me;* wus in front than any place else' "!—And here the jolly doctor laughed till the tears ran down his cheeks in streams a foot deep.

"Reminds me," said the doctor—when he could quit shaking —"reminds me of my old partner, Thompson,—when we were practicing together down at Hog Wallow. He had a case of chill and fever that gave him a lot of trouble. He had done for it about all that could be done, but the chills wouldn't *stay* broke more'n about three weeks. One day we were sitting in the office, criticising Dan'el's last editorial in the *Red Back*, and Thompson was telling about a case he had cured after everybody else had given it up—when in comes his ague case. 'Well, doc.,' says he, with a most woe-begone expression—'I had another one of them shakin' agers yistidy.'

" 'Well, Lorenzo,' said Thompson, throwing himself back with an air, and sticking his thumbs in the arm holes of his vest. "I'll tell you what you do. You know that big spring down back of your house? The run, you know, always keeps up a big damp place there; that is the cause of your chills. It's *malaria*, you know.—Now, you plant *sunflowers* all down that spring branch; sunflowers absorb all the malaria, you know; that will break 'em up sure pop; never knew it to fail!'

" 'Lor! shucks, doc.,' said Lorenzo,—with a cadaverous smile

—'that spring run's been growed up with them sunflowers for *four years* and more;—acres of um!'

"'Damn it,' said Thompson,—'then *cut 'em down!*'"

